# Nanostructure of bioactive glass affects bone cell attachment via protein restructuring upon adsorption

**DOI:** 10.1038/s41598-021-85050-7

**Published:** 2021-03-11

**Authors:** Ukrit Thamma, Tia J. Kowal, Matthias M. Falk, Himanshu Jain

**Affiliations:** 1 Department of Materials Science and Engineering, Lehigh University, Bethlehem, PA 18015 USA; 2 Department of Biological Sciences, Lehigh University, Bethlehem, PA 18015 USA

**Keywords:** Biomaterials, Biomaterials - cells, Biomaterials - proteins, Biomedical materials, Biomineralization

## Abstract

The nanostructure of engineered bioscaffolds has a profound impact on cell response, yet its understanding remains incomplete as cells interact with a highly complex interfacial layer rather than the material itself. For bioactive glass scaffolds, this layer comprises of silica gel, hydroxyapatite (HA)/carbonated hydroxyapatite (CHA), and absorbed proteins—all in varying micro/nano structure, composition, and concentration. Here, we examined the response of MC3T3-E1 pre-osteoblast cells to 30 mol% CaO–70 mol% SiO_2_ porous bioactive glass monoliths that differed only in nanopore size (6–44 nm) yet resulted in the formation of HA/CHA layers with significantly different microstructures. We report that cell response, as quantified by cell attachment and morphology, does not correlate with nanopore size, nor HA/CHO layer micro/nano morphology, or absorbed protein amount (bovine serum albumin, BSA), but with BSA’s secondary conformation as indicated by its β-sheet/α-helix ratio. Our results suggest that the β-sheet structure in BSA interacts electrostatically with the HA/CHA interfacial layer and activates the RGD sequence of absorbed adhesion proteins, such as fibronectin and vitronectin, thus significantly enhancing the attachment of cells. These findings provide new insight into the interaction of cells with the scaffolds’ interfacial layer, which is vital for the continued development of engineered tissue scaffolds.

## Introduction

It is now well established that the nanoscale structure of an engineered tissue scaffold significantly affects its performance, but its mechanistic understanding remains incomplete^1–5^. This is especially the case with multicomponent materials such as bioactive glasses, where the nanoscale structure may show variations of local chemistry even if the average composition and physical morphology appear to be homogeneous. These intertwined variables make the establishment of the role of nanostructure challenging and further complicate the design of optimum materials.

The introduction of nanoscale porosity offers an opportunity to improve scaffold performance by primarily varying physical structure while keeping the composition of the material itself unchanged^3,6–8^. With the introduction of nanopores, the surface area/volume (SA/V) ratio becomes particularly significant as it directly controls the local chemical reactions, including scaffold degradation rate. It is then conceivable that these aspects of the material rather than the nanostructure itself are more directly responsible for the observed impact on cell response. In this context, the bioactivity of a material is often measured by its ability to form hydroxyapatite (HA) and its variants, such as carbonated hydroxyapatite (CHA) on its surface under physiological conditions^4^. With chemical and crystallographic properties similar to that of bone, the formation of a HA/CHA interfacial layer (IL) becomes critical for the recruitment of cells^9^. Additionally, the dissolution products (i.e., soluble silicate and calcium ions) of bioactive glasses can promote cellular response at the gene level, resulting in bone-cell differentiation and maturation and the production of bone matrix^10^. Hence, HA/CHA formation coupled with controlled dissolution makes bioactive glasses a desirable material for promoting hard tissue regeneration. Moreover, surface chemistry and topography of HA have been shown to significantly influence its biological performance^11–13^. Thus, physicochemical differences in materials are likely to dictate their bioactive properties.

Ample research has provided convincing evidence that upon implantation, the surface of bioactive materials is rapidly covered by proteins, and that surrounding cells may not contact or interact with the intrinsic material directly^14^, and that cell/material interactions are rather mediated by a layer comprising of proteins incorporated within the interface of the underlying bioactive substrate^15^. This implies that cells do not detect the nanostructure of a bioactive glass directly and instead are influenced by the proteins that are absorbed^16,17^. Consequently, it is important to understand the dynamics and interactions of the adsorbed proteins and the protein/bioactive material interface itself. Important studies correlate micron-size features and resulting surface roughness of underlying biomaterials with cell response^18–20^. Still, the nature of adsorbed proteins and changes in the surface chemistry potentially resulting from reactions with the local environment require further investigation. In particular, very little is known about how exactly the micro/nano structure of a bioactive glass affects the formation of HA/CHA and/or the structure of absorbed proteins.

Generally, the change in surface topography including nanoporosity leads to concurrent variation of specific surface area, surface charge density, and surface reactivity, which may affect differently the structure of proteins adsorbed on bioactive glass and other biomaterials^21–23^. Recently, we fabricated nanoporous 30 mol% CaO–70 mol% SiO_2_ (30C70S) glass monoliths featuring the same specific surface area but different nanopore sizes (6–44 nm) so that their dissolution characteristics and chemistry were expected to be the same but with different surface nanostructure^24^. These single-variable monoliths permitted the control of HA/CHA formation under a physiological environment, allowing us to unambiguously correlate the topological nanostructure of the underlying nanoporous glass substrate with HA/CHA layer features^24^. Here, in a follow-up study, we investigated the influence of HA/CHA microstructure on MC3T3-E1 pre-osteoblast cell attachment. Furthermore, we examined the nature of adsorbed proteins, including quantity and conformation, on HA/CHA microstructures, which form the interfacial layer between the cells and the underlying biomaterial.

## Materials and methods

### Fabrication of HA/CHA with varying microstructures

**Figure 1:**
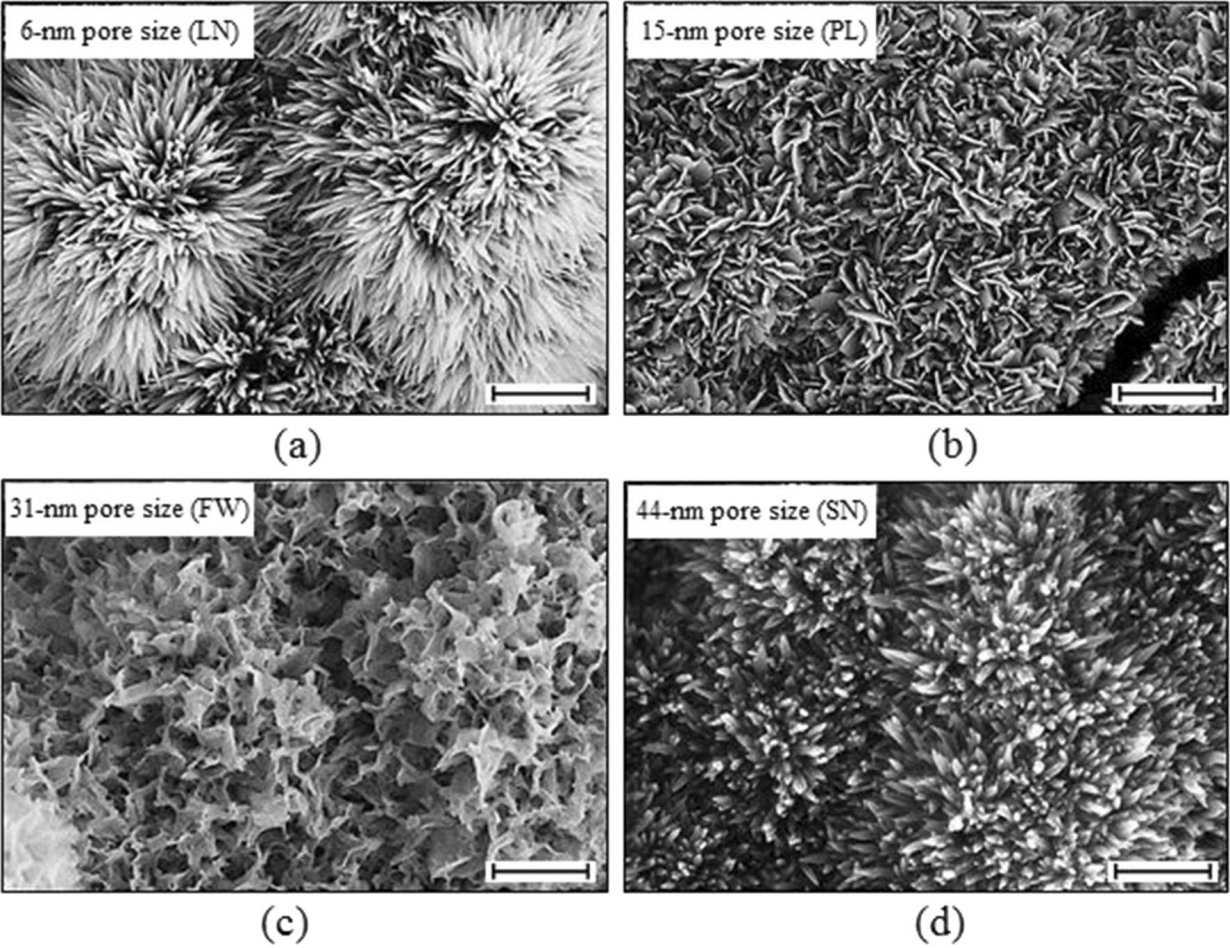
SEM micrographs of HA/CHA layer formed after 3 day PBS incubation on 30C70S nanoporous glass monoliths with different nanopore sizes yet similar specific surface area of 35 ± 2 m^2^/g. Long-needle (LN), plate-like (PL), flower-like (FW), and short-needle (SN) HA/CHA microstructures formed on (**a**) 6 nm, (**b**) 15 nm, (**c**) 31 nm, and (**d**) 44 nm pore-size 30C70S monoliths, respectively (scale bar = 1 µm). Reprinted from “Influence of nanoporosity on the nature of hydroxyapatite formed on bioactive calcium silicate model glass” by Thamma, U., Kowal, T. J., Falk, M. M. & Jain, H. J. Biomed. Mater. Res. Part B 107, p. 893 (2019). Copyright 2018 with permission from John Wiley and Sons.

Four different microstructures of HA/CHA layer on 30 mol% CaO–70 mol% SiO_2_ (30C70S) nanoporous glass monoliths with different nanopore sizes yet similar specific surface area were prepared by incubating monoliths for 3 days in phosphate-buffered saline (PBS) at 37 °C, 5% CO_2_/95% air, and saturated humidity as described previously^24^. This commonly practiced pretreatment is expected to enhance protein adsorption^25^. HA/CHA-covered monoliths with long-needle (LN), plate-like (PL), flower-like (FW), and short-needle (SN) HA/CHA microstructures were obtained on 30C70S nanoporous glass monoliths with 6 nm (a), 15 nm (b), 31 nm (c), and 44 nm (d) median pore sizes but with the same specific surface area of 35 ± 2 m^2^/g (Fig. 1).

### In vitro evaluation of MC3T3-E1 pre-osteoblast attachment

MC3T3-E1 subclone 4 mouse pre-osteoblast cells (American Type Culture Collection, ATCC CRL-2593) were maintained under standard culture conditions (37 °C, 5% CO_2_—95% air atmosphere, saturated humidity) in Alpha-modified Eagles Medium (α-MEM, Gibco/Invitrogen, Grand Island, NY, Cat. # A10490-01) supplemented with 10 vol% fetal bovine serum (FBS) (Atlanta Biologicals, Flowery Branch, GA, Cat. # S11150), 1 vol% penicillin/streptomycin (Corning, Corning, NY, Cat. # 30-001-C1), and 0.06 vol% l-glutamine (HyClone, Logan, UT, Cat. # 25-005-C1). Cells were sub-cultured upon confluency at a 1:10 split ratio. Autoclave-sterilized HA/CHA-covered monoliths placed into 3.5 cm diameter polystyrene cell culture dishes (Corning, Corning, NY, Cat. # 353001) were seeded at a density of 18,000 cell/cm^2^. After 2 h incubation, cells were fixed using 3.7% formaldehyde followed by permeabilization with 0.2% Triton X-100 for 15 min and blocking with 1 (w/v) % BSA diluted in PBS at room temperature for 1 h. Cell nuclei were stained by incubating monoliths in 1xPBS/DAPI (Molecular Probes, Eugene, OR, Cat. # D1306) at 1 µg/ml, and actin filaments were decorated with Alexa488-conjugated phalloidin (Molecular Probes, Cat. # A-12379) at 1:100 dilution at room temperature for 1 h. Monoliths were imaged with a 10 × objective (approximate imaged field = 1 mm^2^) using a Nikon Eclipse TE2000E inverted fluorescence microscope. Cell density was determined by counting and averaging the number of DAPI-stained nuclei per mm^2^. Average cell size was determined by outlining the periphery of individual Alexa488-phalloidin stained cells and measuring the cell area using ImageJ software. Ten images of both nuclei and actin stains were taken from each sample. In order to ensure the stringency of the statistical analyses, four individual specimens for each sample type were processed for every experiment, and the experiments were repeated three times independently.

### Quantification of protein adsorption by Western blotting

The amount of bovine serum albumin (BSA, M_w_ = ~66 kDa), fibronectin (M_w_ = ~220 kDa), and vitronectin (M_w_ = ~75 kDa) adsorbed by the HA/CHA-covered monoliths was quantified by determining the amount of protein depleted from the culture medium using Western blotting. Monoliths were incubated for 2 h in a fully supplemented culture medium at 37 °C, 5% CO_2_/95% air atmosphere, and saturated humidity as described. After incubation, culture media were collected, mixed at a 1:1 volume ratio with SDS sample buffer, incubated at 95 °C for 5 min, and analyzed on 10% SDS–PAGE gels. Gels were electrophoresed at 120 V for 90 min, followed by transfer to nitrocellulose membranes for 90 min at 120 V on ice. After transfer, the membranes were blocked in 5 wt% fat-free dry milk solution prepared in TBST (Tris-buffered saline [TBS] with 1 vol% Tween-20), for 1 h at room temperature, rinsed briefly with TBS to remove the excess blocking solution, and probed with primary antibodies overnight at 4 °C. Antibodies, including anti-BSA rabbit polyclonal (Invitrogen, Eugene, OR, Cat. #A11133) at 1:5000 dilution, anti-fibronectin rabbit polyclonal (Sigma Aldrich, St. Louis, MO, Cat. #F3648) at 1:2000 dilution, and anti-vitronectin mouse monoclonal (ThermoFisher Scientific, Rockford, IL, Cat. #CSI0042702) at 1:1000 dilution, were used. Subsequently, membranes were incubated with secondary antibodies at room temperature for 1 h. BSA and fibronectin were detected with HRP-conjugated goat anti-rabbit antibody (Life Technologies, Carlsbad, CA, Cat. #G21234) at 1:5000 dilution, and Vitronectin was detected with HRP-conjugated goat anti-mouse antibody (Invitrogen, Eugene, OR, Cat. #G21040) at 1:5000 dilution. Purified BSA (Sigma Aldrich, St. Louis, MO, Cat. #A7906), bovine-plasma-derived fibronectin (Sigma Aldrich, St. Louis, MO, Cat. #F4759), and bovine-plasma-derived vitronectin (R&D Systems, Minneapolis, MN, Cat. #2348VN) were analyzed as controls. Proteins were detected using X-ray film and Enhanced Chemiluminescent (ECL) reagent, and densitometry was performed using ImageJ software. The amount of proteins was calculated from a standard curve constructed by a series of known concentrations of the purified protein. In order to ensure the reliability of the data, experiments were repeated three times with three different sets of samples on separate occasions.

### Characterization of adsorbed BSA conformations on HA/CHA layer by ATR-FTIR

Attenuated total reflection Fourier transform infrared (ATR-FTIR) spectroscopy was performed in order to characterize the conformation of BSA absorbed onto HA/CHA-covered monoliths. HA/CHA-covered monoliths were incubated with 10 mg/ml BSA in 1xPBS solution for 2 h under standard culture conditions. After incubation, samples were gently rinsed with 1xPBS in order to remove unbound BSA from its surface, followed by drying at 37 °C in ambient atmosphere. Lyophilized BSA, HA/CHA-covered monoliths (without BSA incubation), and BSA-coated HA/CHA-covered monoliths were characterized by ATR-FTIR spectroscopy using a Bruker Vertex 70 spectrometer (Bruker Optics, Billerica, MA) equipped with a mercury cadmium telluride (MCT) detector and an MVP Pro single reflection diamond crystal ATR accessory (Harrick Scientific, Pleasantville, NY). Spectra were acquired in the mid-infrared range of 650–4000 cm^−1^ wavenumbers by averaging 400 scans at 6 cm^−1^ resolution and were reported in absorbance units. In order to characterize the conformation of BSA adsorbed on HA/CHA-covered monoliths, the ATR-FTIR spectra of BSA-coated monoliths were subtracted by those without BSA incubation and then deconvoluted using Gaussian function.

### Statistical analyses

For the quantifications of MC3T3-E1 pre-osteoblast attachment and protein adsorption, one-tailed, unpaired t-tests were performed in Microsoft Excel to determine statistical significance where a *p* value < 0.05 was considered to be statistically significant.

## Results

### Attachment of MC3T3-E1 cells on HA/CHA layer with different microstructures

**Figure 2:**
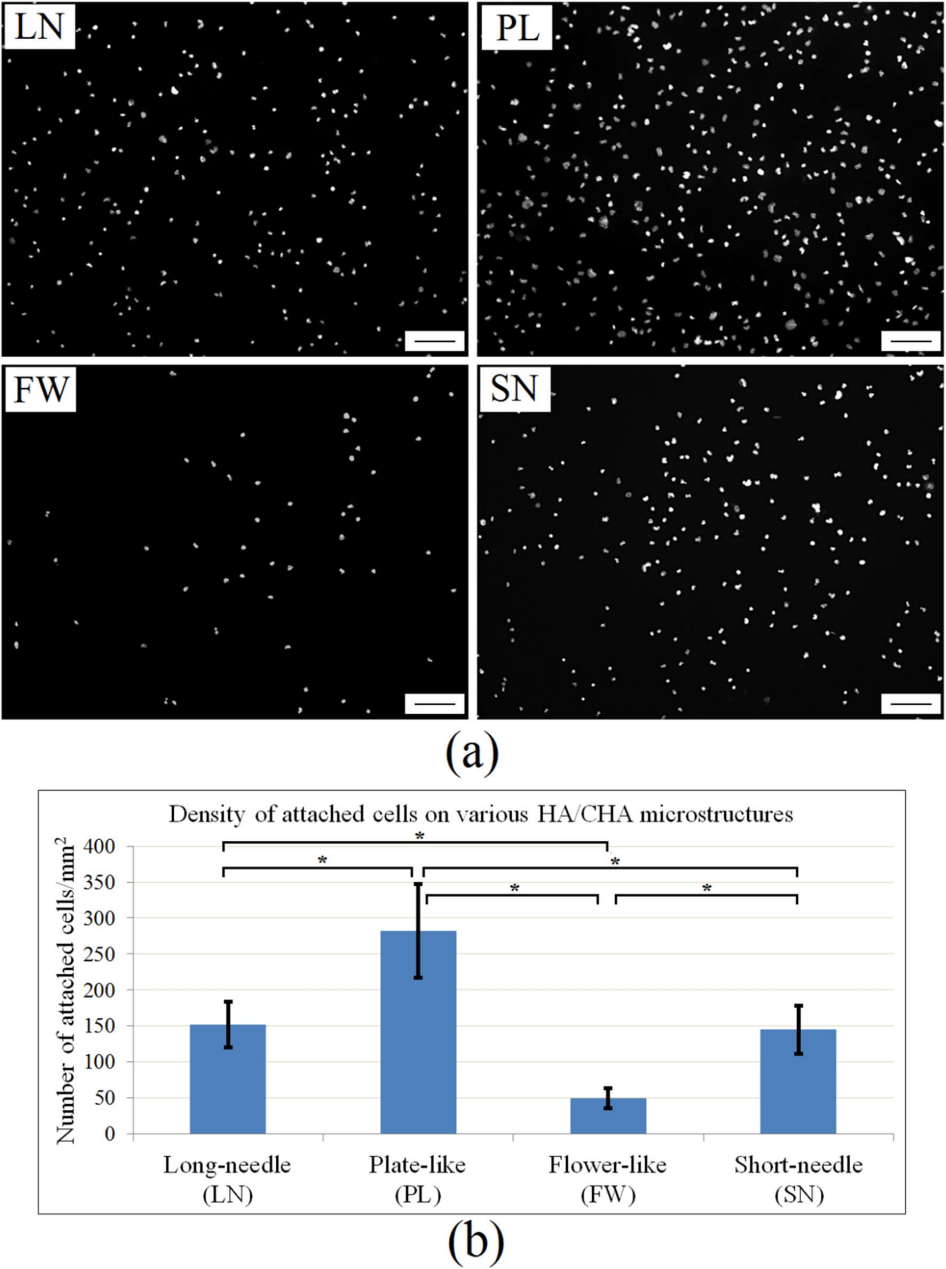
Number of cells attached to bioactive glass monoliths with different nanopore size and HA/CHA microstructure. Cell nuclei of MC3T3-E1 cells attached after 2 h to monoliths were stained with DAPI and imaged by fluorescence microscopy. (**a**) Representative black and white images of nuclei of attached cells on long-needle (LN), plate-like (PL), flower-like (FW), and short-needle (SN) HA/CHA-covered monoliths are shown (scale bar = 100 µm). (**b**) Density of cells attached onto the HA/CHA layer quantitatively determined by counting the number of cell nuclei per mm^2^ on multiple representative images/monolith type in three independent experiments. Significant differences in cell numbers are marked with *.

30C70S nanoporous glass monoliths that differed only in nanopore size formed distinct HA/CHA microstructures after 3 day incubation in 1xPBS solution. Long-needle (LN), plate-like (PL), flower-like (FW), and short-needle (SN) HA/CHA crystals were observed on monoliths with 6 nm, 15 nm, 31 nm, and 44 nm pore size, respectively (Fig. 1)^24^. MC3T3-E1 pre-osteoblasts (bone-forming cells), widely used as a model for studying in vitro biological performance of biomaterials for hard tissue applications^26,27^, were seeded onto these HA/CHA-covered monoliths. Two hours after cell seeding, the nuclei of cells attached to LN, PL, FW, and SN microstructures were stained with DAPI and visualized by fluorescence microscopy (Fig. 2a). In order to quantify cell attachment, cell nuclei were counted to determine the number of cells attached onto each HA/CHA microstructure. The average densities of attached cells on LN, PL, FW, and SN microstructures were 152 ± 32 cells/mm^2^, 282 ± 65 cells/mm^2^, 49 ± 14 cells/mm^2^, and 145 ± 33 cells/mm^2^, respectively (Fig. 2b).

**Figure 3:**
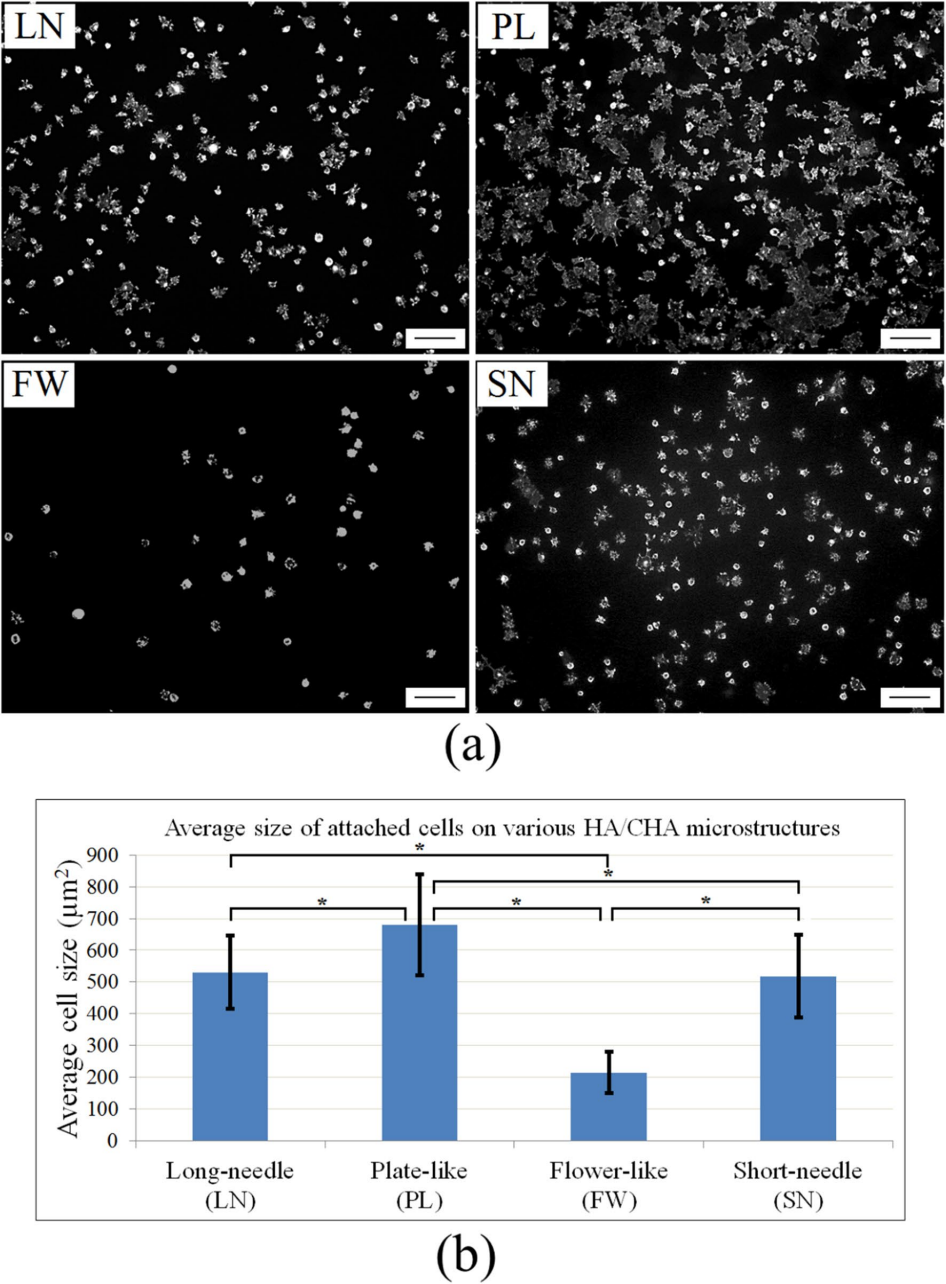
Size of cells attached to bioactive glass monoliths with different nanopore size and HA/CHA microstructure. The actin cytoskeleton of MC3T3-E1 cells attached after 2 h to monoliths was stained with Alexa488-conjugated phalloidin and imaged by fluorescence microscopy. (**a**) Representative black and white images of the actin cytoskeleton of cells attached on long-needle (LN), plate-like (PL), flower-like (FW), and short-needle (SN) HA/CHA-covered monoliths (scale bar = 100 µm). (**b**) Quantitative analyses of the average size of attached cells on the HA/CHA layer determined by measuring the stained area covered by cells in multiple images of three independent experiments. Significant differences in cell numbers are marked with *.

In order to determine the average size of attached cells, the actin cytoskeleton of cells growing for 2 h on LN, PL, FW, and SN microstructures was stained with Alexa488-conjugated phalloidin. The total area covered by the actin cytoskeleton was measured (Fig. 3a), and then divided by the number of attached cells. The calculated average size of attached cells on LN, PL, FW, and SN microstructures was 531 ± 115 µm^2^, 680 ± 159 µm^2^, 215 ± 66 µm^2^, and 518 ± 130 µm^2^, respectively (Fig. 3b). Based on cell density and average cell size, cells preferred to attach and spread out the most on the HA/CHA plate-like (PL) microstructure. On average, approximately six times more cells attached and spread out three times more on the PL microstructure compared to the least preferred flower-like (FW) HA/CHA microstructure, whereas cells exhibited comparable levels of preference for the LN and SN HA/CHA microstructures in between the two extremes.

### Quantification of protein adsorption

Quantitative analyses of fibronectin, vitronectin, and BSA adsorbed by the four different HA/CHA microstructures shown in Fig. 1 were carried out using Western blotting. In order to quantify the concentration of adsorbed proteins, experiments were designed to measure the amounts of these proteins depleted from the culture medium after 2 h of incubation in the presence of HA/CHA-covered monoliths rather than measuring absorption directly. This strategy avoided the difficulties associated with reliably extracting the adsorbed proteins from the crystalline HA/CHA-covered surface. Densitometry of the Western blots was carried out using ImageJ software, and the concentrations of proteins were calculated from standard curves constructed using known concentrations of purified proteins.

**Figure 4:**
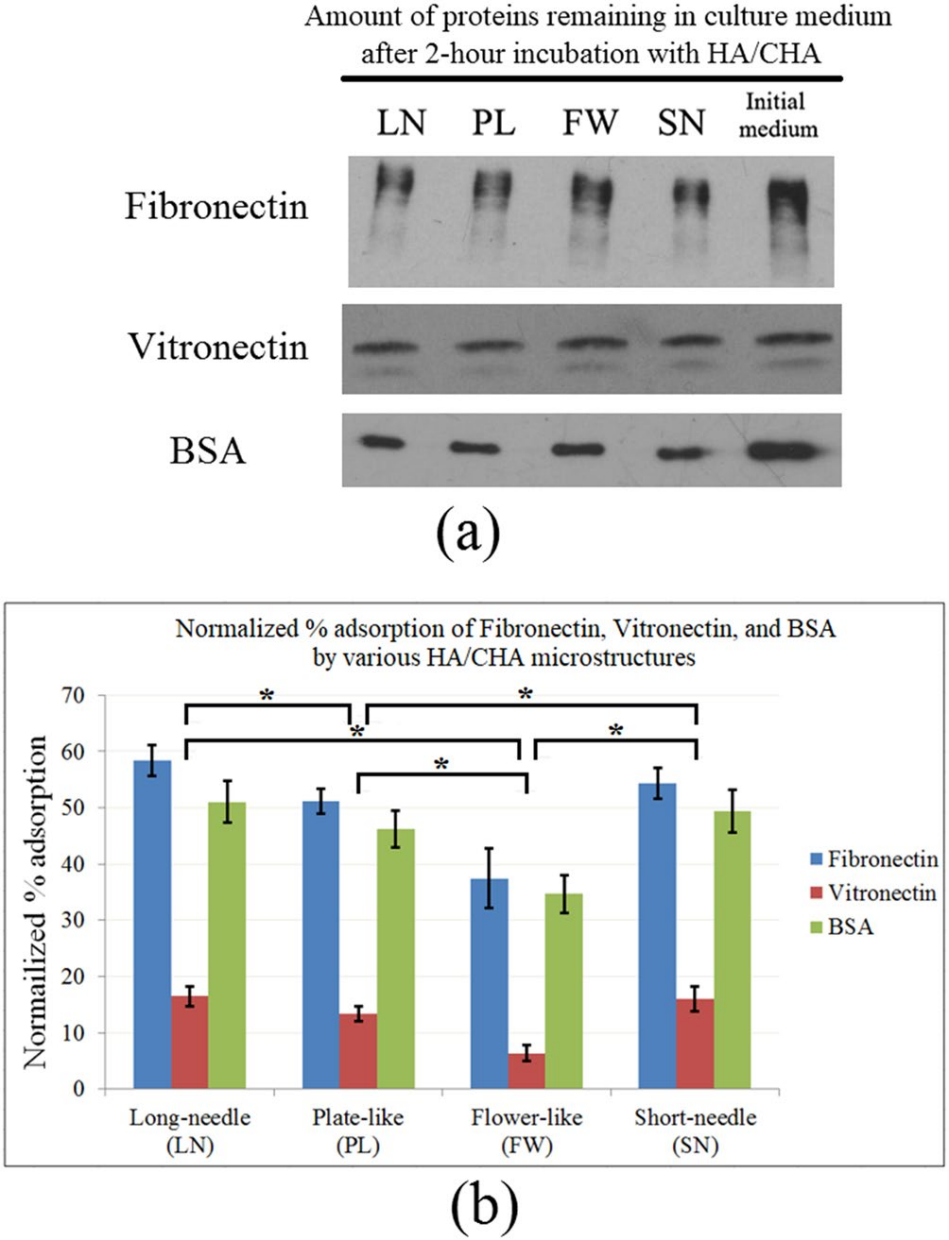
Quantitative analysis of proteins in culture medium adsorbed by HA/CHA. (**a**) Western blots of Fibronectin, Vitronectin, and BSA remaining in culture medium after a 2 h incubation period in the presence of HA/CHA-covered monoliths with four HA/CHA microstructures. (**b**) Average normalized % adsorption of Fibronectin (blue), Vitronectin (red), and BSA (green) on HA/CHA-covered monoliths with four HA/CHA microstructures. Significant differences in cell numbers are marked with *.

**Figure 5:**
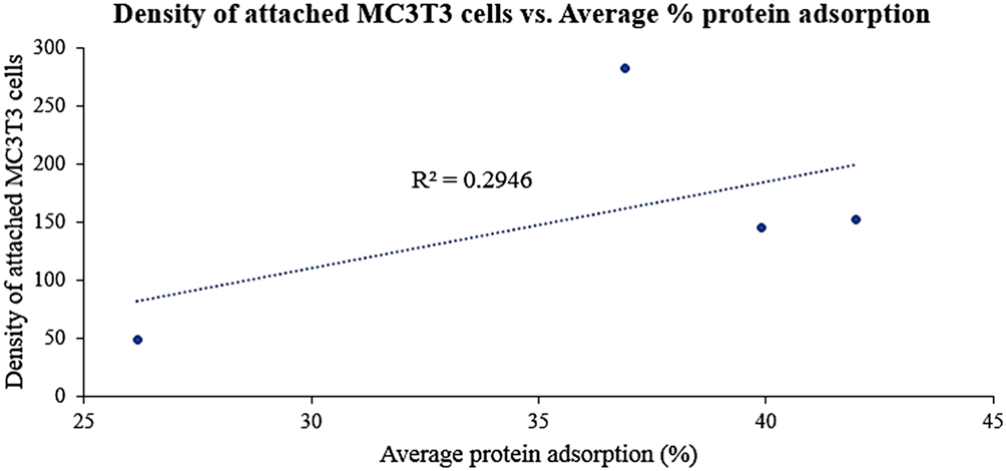
Density of attached MC3T3-E1 cells plotted as a function of average % adsorption of all three culture medium proteins: Fibronectin, Vitronectin, and BSA.

Representative Western blots of the culture medium detecting fibronectin, vitronectin, and BSA after 2 h incubation in the presence of HA/CHA-covered monoliths are shown in Fig. 4a. The intensities of fibronectin, vitronectin, and BSA bands were noticeably weaker than those found in the initial culture medium, suggesting that all three proteins were absorbed readily on all HA/CHA microstructures. The % adsorption was calculated through a normalization of the depleted amounts of proteins by those amounts present in the initial culture medium (Fig. 4b). The protein adsorptions by HA/CHA microstructures exhibit a comparable trend for all three proteins, where the highest to lowest % adsorption of each protein is found on LN and SN comparatively, followed by PL and then FW. In order to determine the correlation between cellular performance and protein adsorption, the density of attached MC3T3-E1 cells determined as described in “Attachment of MC3T3-E1 cells on HA/CHA layer with different microstructures” section has been plotted in Fig. 5 as a function of the average % adsorption of fibronectin, vitronectin, and BSA. The R^2^ value of the linear trendline for this plot is only 0.2946, indicating that cell attachment does not correlate closely with the amount of adsorbed fibronectin, vitronectin, or BSA.

### Characterization of BSA conformation on HA/CHA layer

The characterization of protein conformation on the different HA/CHA layers was carried out by attenuated total reflection Fourier transform infrared (ATR-FTIR) spectroscopy using bovine serum albumin (BSA) as a representative model protein. It is by far the most abundant protein constituent present in FBS-supplemented cell culture medium (~ 61%)^28–31^ and therefore was chosen as the model for the study of protein conformation on HA/CHA. The different HA/CHA-covered monoliths were incubated with 10 mg/ml BSA solution under standard culture conditions for 2 h. The FTIR spectra of lyophilized BSA, HA/CHA-covered monoliths (without BSA incubation), and BSA-coated HA/CHA-covered monoliths were acquired.

**Figure 6:**
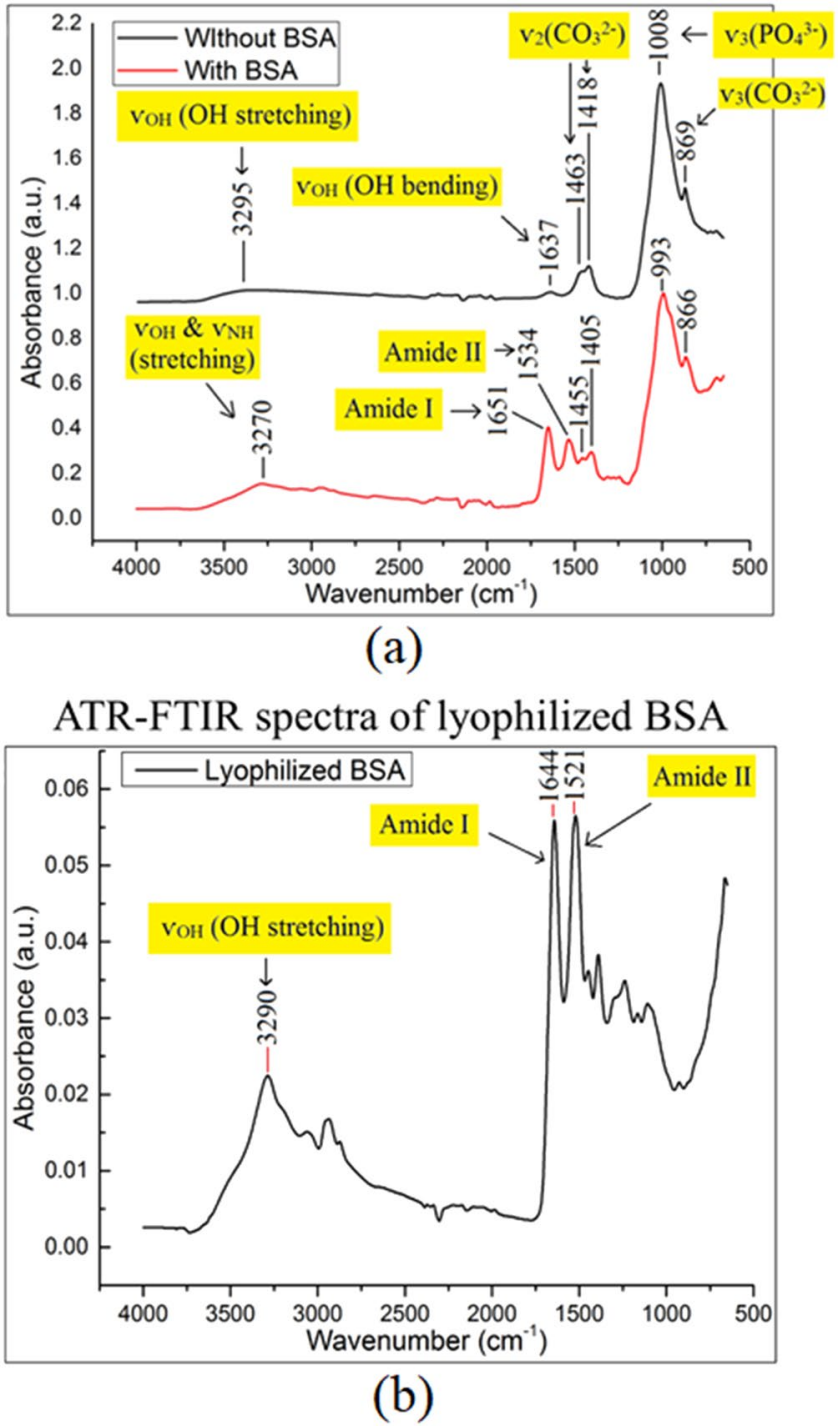
(**a**) Representative ATR-FTIR spectra of long-needle (LN) HA/CHA samples with (red) and without (black) 2-h BSA incubation. (**b**) Representative ATF-FTIR spectrum of lyophilized BSA acquired in control.

Representative ATR-FTIR spectra of LN HA/CHA samples with and without BSA incubation are shown in Fig. 6a. Without BSA incubation, six characteristic absorbance peaks are observed on all HA/CHA microstructures at: 3289 ± 15 cm^−1^, 1637 ± 5 cm^−1^, 1463 ± 5 cm^−1^, 1418 ± 5 cm^−1^, 1008 ± 7 cm^−1^, and 869 ± 4 cm^−1^. The absorbance peaks at 3289 cm^−1^ and 1637 cm^−1^ are attributed to the stretching vibration and the bending vibration of OH groups, respectively, which are due to the presence of HA/CHA and adsorbed water molecules^32,33^. The absorbance peak at 1008 cm^−1^ corresponds to the antisymmetric stretching vibration (ѵ_3_) of PO_4_^3−^ groups in HA/CHA and NaH_2_PO_4_^34^. The peaks at 1463 cm^−1^ and 1418 cm^−1^ are attributed to asymmetric stretching vibration (ѵ_3_) of CO_3_^2−^, while the peak at 869 cm^−1^ is attributed to the bending vibration (ѵ_2_) of CO_3_^2−^ groups^35^. Notably, ATR-FTIR spectra of BSA-coated HA/CHA monoliths for all microstructures show a set of additional peaks at 3277 ± 5 cm^−1^, 1648 ± 3 cm^−1^, and 1530 ± 4 cm^−1^ compared to the spectra of samples without BSA incubation. These three additional peaks correlate with the presence of BSA, which possesses corresponding characteristic absorbance peaks at 3290 cm^−1^, 1644 cm^−1^, and 1521 cm^−1^ (see Fig. 6b). The characteristic peak at 3290 cm^−1^ is associated with the stretching vibration of NH groups, while those at 1644 cm^−1^ and 1521 cm^−1^ are attributed to Amide I and Amide II vibration, respectively^36,37^. Amide I vibration, absorbing infrared in the 1600–1700 cm^−1^ frequency range, arises predominantly from the stretching vibrations of C=O and C–N groups, which link the amino acids within the protein backbone structure^38,39^. The frequencies in the Amide I region are highly sensitive to the protein secondary structure. On the other hand, Amide II vibration, absorbing infrared in the 1510–1590 cm^−1^ frequency range, originates mainly from the in-plane vibration of NH groups, resulting in less sensitivity to the protein secondary structure compared to its Amide I counterpart^38^. Therefore, our characterization of BSA protein conformation is mainly based on the Amide I region.

**Figure 7:**
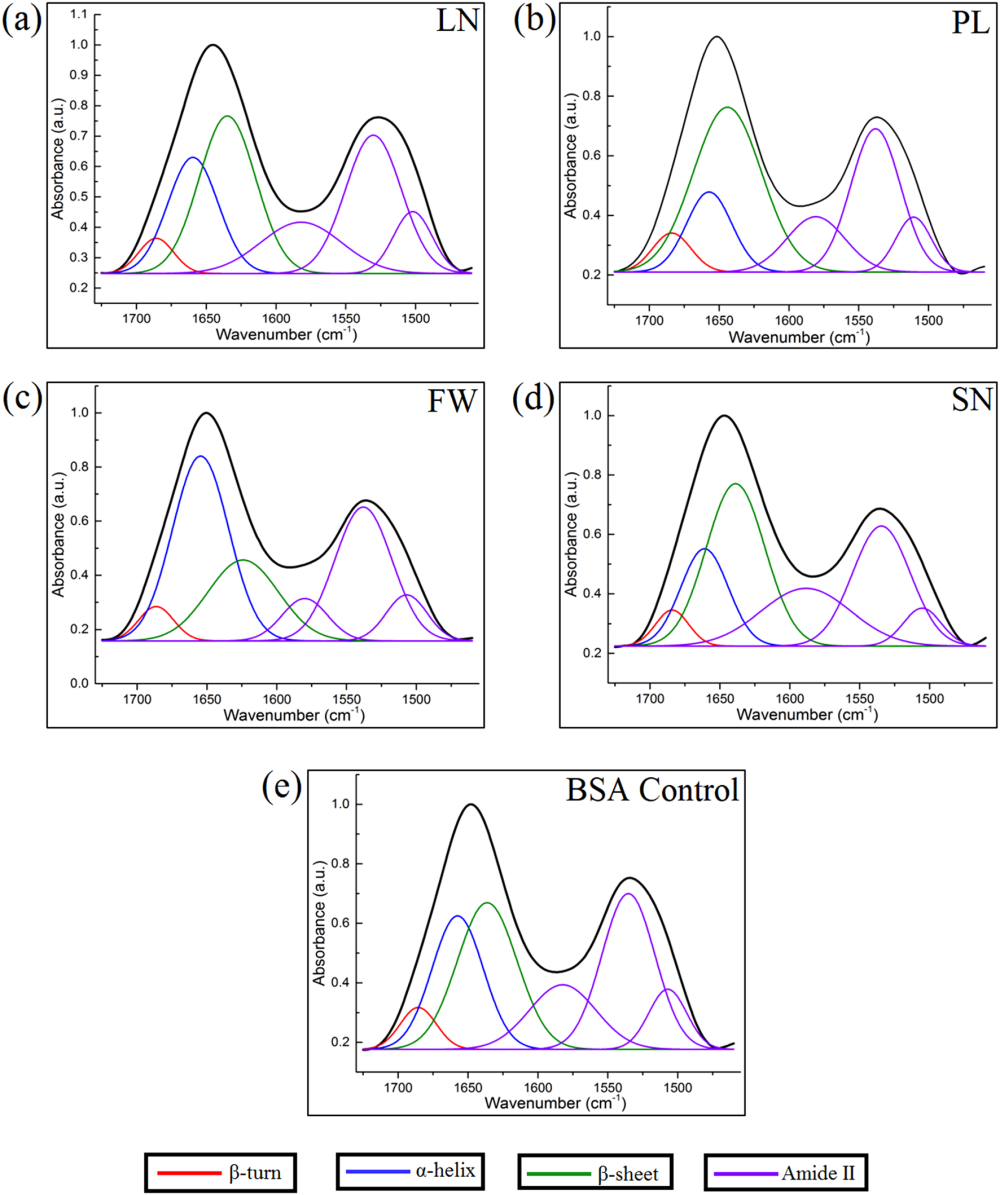
Deconvolution of the amide region of FTIR spectrum of adsorbed BSA on HA/CHA samples with (**a**) long-needle (LN), (**b**) plate-like (PL), (**c**) flower-like (FW), and (**d**) short-needle (SN) microstructures and in (**e**) of lyophilized BSA.

In order to characterize the conformation of BSA adsorbed onto the different HA/CHA microstructures, the ATR-FTIR spectra of BSA adsorbed to HA/CHA-covered monoliths were subtracted by those without BSA incubation and were then deconvoluted using Gaussian function. In order to maintain a consistent baseline correction of subtracted spectra, Amide II vibrations were included in the deconvolution but were not applied in the analysis of protein conformation due to their poor sensitivity, as previously mentioned^38^. The deconvoluted spectra in Amide I and Amide II regions of adsorbed BSA on HA/CHA samples with LN, PL, FW, and SN microstructures and lyophilized BSA are shown in Fig. 7a–e, respectively. The peak positions, full-width-at-half-maximum (FWHM), and % relative areas of β-turn, α-helix, and β-sheet structural motifs in Amide I of adsorbed BSA on various HA/CHA microstructures and lyophilized BSA are summarized in Table 1. The peak positions of β-turn, α-helix, and β-sheet structures in Amide I found in adsorbed BSA and lyophilized BSA are in agreement with the values reported in the literature^39–42^. Briefly, related to Amide I, the components at 1690–1670 cm^−1^ were assigned to the β-turn structure, the ones at 1660–1650 cm^−1^ were assigned to the α-helix structure, and those at 1640–1620 cm^−1^ were assigned to the β-sheet structure. The β-turn relative contents were comparable for all HA/CHA microstructures, and there was no significant difference relative to lyophilized BSA as well. Interestingly, in comparison to lyophilized BSA, a decrease in α-helix and an increase in β-sheet relative fractions were observed for adsorbed BSA on HA/CHA with LN, PL, and SN microstructures. This implies that BSA was less folded after being absorbed on LN, PL, and SN microstructures due to a conversion from a rigid helical structure to an extended sheet structure^43–45^. In contrast, higher α-helix fraction and lower β-sheet fraction compared to lyophilized BSA were observed on adsorbed BSA on HA/CHA with FW microstructure, indicating that BSA became more folded after being absorbed on FW microstructure^43,45^.

**Table 1:** Summary of peak positions, full-width-at-half-maximum (FWHM) values, and % relative areas of β-turn (underlined), α-helix (italicized), and β-sheet (bolded) structural motifs in the Amide I region of BSA absorbed on various HA/CHA microstructures and in control on lyophilized BSA.

Peak position (cm^−1^)	FWHM (cm^−1^)	Relative area (%)	Peak Assignment
**Long-needle (LN)**			
1686	29	4.0	β-turn
*1659*	*42*	*19.3*	*α-helix*
**1635**	**48**	**29.4**	**β-sheet**
**Plate-like (PL)**			
1684	32	5.2	β-turn
*1657*	*38*	*12.6*	*α-helix*
**1639**	**58**	**39.3**	**β-sheet**
**Flower-like (FW)**			
1686	30	4.2	β-turn
*1654*	*47*	*36.2*	*α-helix*
**1627**	**59**	**19.8**	**β-sheet**
**Short-needle (SN)**			
1684	28	4.1	β-turn
*1661*	*40*	*15.9*	*α-helix*
**1639**	**49**	**33.0**	**β-sheet**
**Lyophilized BSA**			
1686	30	4.7	β-turn
*1657*	*43*	*21.4*	*α-helix*
**1636**	**50**	**27.3**	**β-sheet**

**Figure 8:**
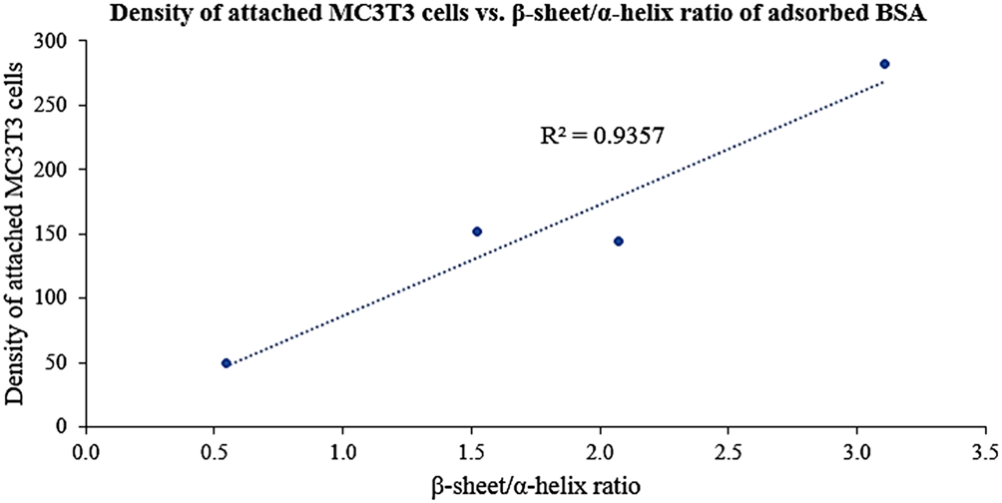
Density of attached MC3T3-E1 cells versus the β-sheet/α-helix ratio of adsorbed BSA.

Furthermore, the β-sheet/α-helix ratio, a measure for the degree of BSA unfolding^44^, for adsorbed BSA on each HA/CHA-covered sample, was calculated for each microstructure. Its value for LN, PL, FW, and SN microstructures is 1.52, 3.11, 0.55, and 2.07, respectively. The highest to the lowest degree of BSA unfolding was found on HA/CHA with PL, SN, LN, and FW microstructures, respectively. In order to determine the level of correlation between cellular performance and conformation of adsorbed BSA, the density of attached MC3T3-E1 cells determined as described in “Attachment of MC3T3-E1 cells on HA/CHA layer with different microstructures” section is plotted in Fig. 8 as a function of the β-sheet/α-helix ratio of adsorbed BSA. The R^2^ value of the linear trendline for this plot is 0.9357, which clearly shows a positive correlation of BSA conformation with the level of initial MC3T3-E1 cell attachment.

## Discussion

Hydroxyapatite/carbonated-hydroxyapatite (HA/CHA) with long-needle (LN), plate-like (PL), flower-like (FW), and short-needle (SN) microstructures were successfully fabricated on 30C70S nanoporous glass monoliths by varying nanopore size (Fig. 1), while keeping all other variables, including specific surface area, unchanged. Since the local chemical environment is effectively established by the surface area of monoliths, the samples differed primarily in the microstructure of the HA/CHA layer. MC3T3-E1 pre-osteoblast cells were used as a model to characterize the biological response to these HA/CHA microstructures. Based on the density and average size of attached cells, the most preferred HA/CHA microstructure was PL, and the least preferred microstructure was FW, whereas the needle-like microstructures, LN and SN, show a comparable level of cell preference between the two extremes. From this observation, one may directly connect the differences in cell response to the topographical variations of HA/CHA microstructures. However, as PL and FW morphologies are more similar to each other compared to needle-like morphologies, cell attachment and adhesion do not simply correlate with HA/CHA morphology. In addition, as physiological solutions come into contact with the surface of biomaterials, proteins are spontaneously adsorbed onto the biomaterial surface, long before cell attachment, proliferation, or differentiation occurs^46^. Thus, cells are unlikely to interact directly with the surface of the substrate, but rather with the protein layer deposited onto the surface of biomaterials^47,48^. In other words, the properties of the underlying substrate may not impact the biological response of cells directly, but they may do so indirectly through the adsorbed protein layer, which ultimately guides cell behavior on the biomaterials^49–51^. Therefore, in order to explain the differences in cell attachment and adhesion on different HA/CHA microstructures, it is advantageous to understand how the amount and structure/conformation of adsorbed proteins is affected by HA/CHA microstructures, which, in our case, are determined by the nanopore size of the 30C70S bioactive glass substrate.

Bovine serum albumin (BSA), fibronectin, and vitronectin, essential proteins present in blood serum and lymph fluids, were chosen as models to study the protein adsorption on the four different HA/CHA microstructures. The extracellular matrix (ECM) glycoproteins, fibronectin and vitronectin, present in blood serum have been shown to play a vital role in facilitating cell attachment on biomaterials due to the presence of arginine-glycine-aspartic acid (RGD) motifs in their polypeptide sequences to which cellular integrin adhesion receptors bind^52^. As FBS in essence is the serum component of blood, fibronectin and vitronectin are also present in FBS-supplemented cell culture medium used in this study. While fibronectin and vitronectin are present at low concentrations of 0.07% and 0.11%, respectively^30,31^, BSA makes up the vast majority (~ 61%) of all proteins in the FBS-supplement^28,29,31^. Although BSA does not contain RGD sequence motifs, it is likely to be readily adsorbed by HA/CHA layer due to its vast abundance and to play an indirect role in cell attachment^53,54^. FBS contains additional proteins, however none are abundant^28–31^, and, to our knowledge, only fibronectin and vitronectin harbor RGD cell attachment motifs. Yet, we cannot exclude the possibility that another adsorbed serum protein contributes to our observed cell response.

**Figure 9:**
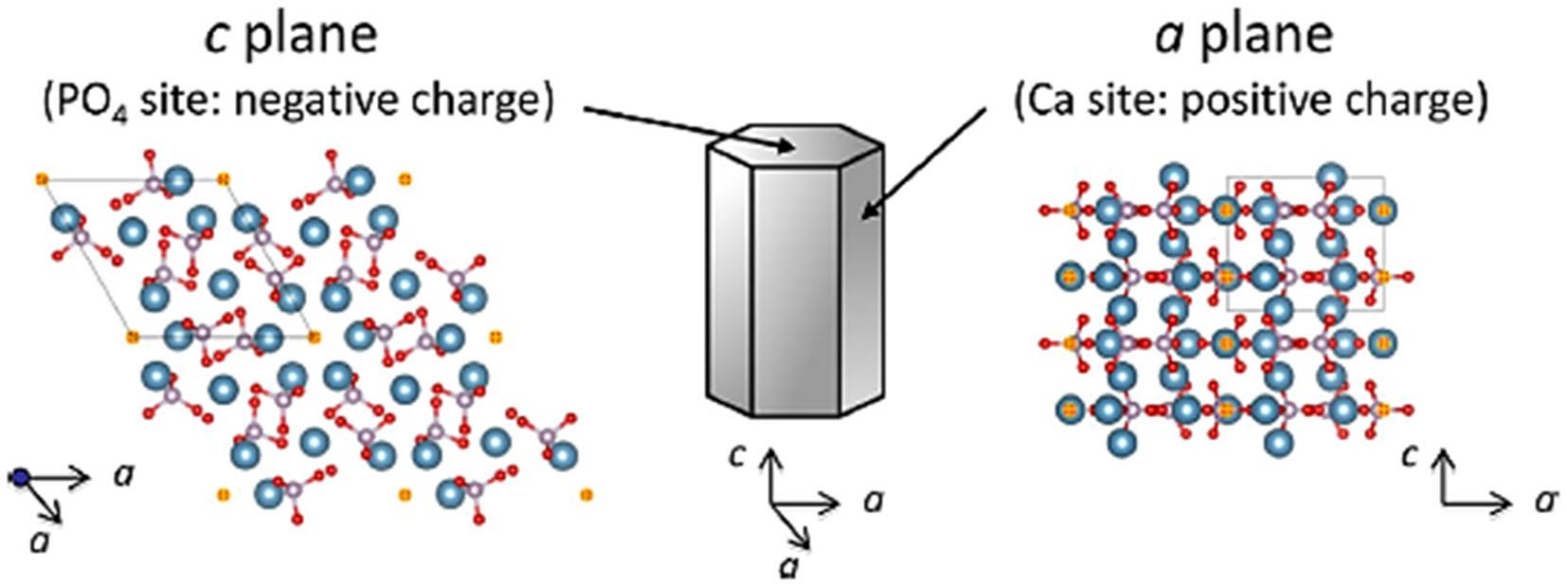
Schematic illustration of hydroxyapatite (HA) crystal structure consisting of positively charged *a* plane and negatively charged *c* plane. The blue, white, red, and yellow spheres represent Ca, P, O, and OH, respectively. Reprinted from “Synthesis and modification of apatite nanoparticles for use in dental and medical applications” by Okada, M. & Matsumoto, T. *Jpn. Dent. Sci. Rev.* **51**, p. 87 (2015). Copyright 2015 with permission from Elsevier.

The difference in % proteins adsorption is thought to be caused by the differences in net surface charge of the HA/CHA microstructures^21,55,56^. HA/CHA belongs to a hexagonal crystal structure that possesses two major types of crystal planes: *a* plane and *c* plane, as illustrated in Fig. 9^57^. The *a* plane, rich in Ca^2+^ sites, is positively charged, whereas the *c* plane, rich in PO_4_^3−^ and OH^−^ sites, is negatively charged. The needle-like HA/CHA microstructure is a result of HA/CHA formation under a slow, near-equilibrium condition where HA/CHA crystals predominantly grow along the *c*-axis with a much slower growth rate along the *a*-axis direction^24,58,59^. Thus, the dominating surface of the needles is the cylindrical surface made of *a* planes. With increasing HA/CHA growth rate under kinetics-limited growth condition, HA/CHA crystals are more likely to grow in a single *a*-axis direction, yielding plate-like HA/CHA crystals, or multiple *a*-axis directions, producing flower-like HA/CHA crystals, respectively^24^. The dominating surface of PL and FW microstructures is relatively flat and close to *c* plane orientation. Hence, needle-like (LN and SN) HA/CHA microstructures with a larger relative ratio of *a*-plane/*c*-plane area exhibit an overall more positively charged surface compared to PL and FW HA/CHA microstructures. This variation in net surface charge of different HA/CHA microstructures is further supported by observations made by Zhuang and Aizawa^60^, who reported a more positive surface zeta potential of rod-shaped HA, exhibiting the dominant presence of *a* plane surface, compared to that of plate-shaped HA that is dominated by *c* planes. Consequently, HA/CHA with PL microstructure would exhibit a more positively charged surface compared to the FW microstructure due to a relatively larger *a*-plane/*c*-plane area ratio. In short, the most to the least positively charged HA/CHA microstructures are in the order of LN–SN, PL, and FW, respectively. Because fibronectin, vitronectin, and BSA are all acidic proteins with isoelectric points of 5.9, 5.7, and 4.7, respectively in culture medium at pH 7.4^61,62^, they have a net negative charge and are likely to be adsorbed predominantly on the surface that is more positively charged due to electrostatic attraction. Hence, the highest to lowest absorption of these three proteins on HA/CHA is in the order of needle-like (LN ~ SN) > PL > FW microstructures, respectively, which is in good agreement with the present experimental results of protein adsorption. A similar observation to ours that surface charge influences protein adsorption due to electrostatic interaction was also made by Zhuang and Aizawa^60^, who reported that BSA absorbed more on rod-shaped HA compared to plate-shaped HA, while the opposite effect was observed for lysozyme, a basic protein, due to a more positive zeta potential of rod-shaped HA compared to that of plate-shaped HA.

However and more importantly, amounts of adsorbed fibronectin, vitronectin, and BSA do not simply correlate with the level of cell attachment observed on our HA/CHA microstructures, demonstrating that cell-substrate interaction is not governed by the amounts of fibronectin, vitronectin, or BSA adsorbed on the HA/CHA layer alone. This lack of correlation is consistent with other studies such as by Budd et al.^63^ and Steele et al.^64^, who found that although the presence of fibronectin and vitronectin in the culture medium is necessary for cell attachment, cell attachment only requires a certain minimum concentration of each protein, and any extra amount beyond the required concentrations is not accompanied by increased cell attachment and/or cell spreading. So the question arises: what governs cell-attachment preferences to the different HA/CHA microstructures?

Though BSA is not believed to play a direct role in facilitating cell attachment, our ATR-FTIR results demonstrate a positive correlation between the β-sheet fraction of adsorbed BSA and the level of MC3T3-E1 attachment on our HA/CHA microstructures. The degree of protein unfolding may be quantified by the β-sheet/α-helix ratios since β-sheets represent an extended structure while α-helices correspond to the rigid helical structure of BSA^44^. As the β-sheet/α-helix ratio increases, the density and average size of attached cells increase, suggesting that the unfolding of BSA upon adsorption enhances initial cell attachment. This might be because the β-sheet structure in BSA interacts with and triggers the ‘active’ conformation of RGD-containing adhesion proteins upon adsorption. A similar observation is reported by Koblinski et al., who found that non-adhesive proteins, like BSA, interact with fibronectin at low concentrations during the adsorption process and activate the RGD sequence, resulting in enhanced cell attachment^65^. However, it is not fully clear how HA/CHA microstructures influence the conformation of adsorbed BSA. Since the Ca^2+^ and PO_4_^3−^ ions are believed to be the protein-binding sites on HA/CHA surfaces providing a major driving force for protein adsorption^66^, a possible explanation is that the variations in the Ca^2+^/PO_4_^3−^ ratios can attract different functional BSA polypeptide groups, leading to different conformations of BSA absorbed on various HA/CHA microstructures.

## Conclusions

To begin answering the fundamental question of what cells perceive when attaching to the surface of bioactive glasses, we fabricated and tested sol-gel-derived nanoporous monoliths with four different nanopore sizes. The chemical composition and surface area of the starting 30C70S monoliths were identical, thus establishing the same chemical and physical characteristics except for nanoscale pore size. The difference in nanopores alone led to four distinct HA/CHA microstructures, viz. long needles (LN), plates (PL), flowers (FW), and short needles (SN). A positive correlation is found between the amount of tested proteins (BSA, fibronectin, vitronectin) and the overall area of positively charged *a*-planes of HA crystal structure, which decreases in the order LN > SN > PL > FW microstructures.

Among the four distinct HA/CHA microstructures a clear preference of MC3T3-E1 pre-osteoblast cells was found for attaching and spreading out on the PL microstructure. The FW microstructure, while similarly shaped like PL, demonstrated the lowest level of cell attachment. These results show that cells respond to interfacial layer characteristics that are a consequence of scaffold micro/nano structure. Furthermore, cell attachment did not correlate with the size of the monolith nanopores or the amount of the three proteins that adsorbed from the cell culture medium onto the HA/CHA microstructures, but with BSA’s secondary conformation, specifically the β-sheet/α-helix ratios in the Amide I region of BSA. It remains to be determined how such secondary structure of proteins is communicated to and incorporated within the subcellular mechanisms responsible for a cell’s attachment, proliferation and differentiation to an engineered substrate.

## Supplementary Information

Supplementary Information.

## Data Availability

The datasets generated during and/or analyzed during the current study are not publicly available due to technical and time limitations but are available from the corresponding author on reasonable request.
