# Supplementary material for: Nanostructure of bioactive glass affects bone cell attachment via protein restructuring upon adsorption
Source: Sci Rep. 2021 Mar 11;11:5763. doi: 10.1038/s41598-021-85050-7 (PMC7952393; doi:10.1038/s41598-021-85050-7)
Supplement: Supplementary file 1 — Supplementary Information. [file 41598_2021_85050_MOESM1_ESM.docx]

**Supplemental Data**

Nanostructure of Bioactive Glass Affects Bone Cell Attachment via Protein Restructuring upon Adsorption

Ukrit Thamma^1^, Tia Kowal^2^, Matthias Falk^2^, Himanshu Jain^1,*^

^1^Department of Materials Science and Engineering, Lehigh University, Bethlehem, PA 18015, USA

^2^Department of Biological Sciences, Lehigh University, Bethlehem, PA 18015, USA

* Corresponding author

**Running title:** Role of nanostructure on the biological response of bone cells

**Key words:** Nanostructured bioactive glass; hydroxyapatite morphology; cellular response; protein adsorption; protein conformation

________________________________________________

***Address correspondence to:**

Himanshu Jain

Department of Materials Science & Engineering

Lehigh University

Bethlehem, PA 18015

Email: H.Jain@Lehigh.edu

Phone: 001-610-758-4217


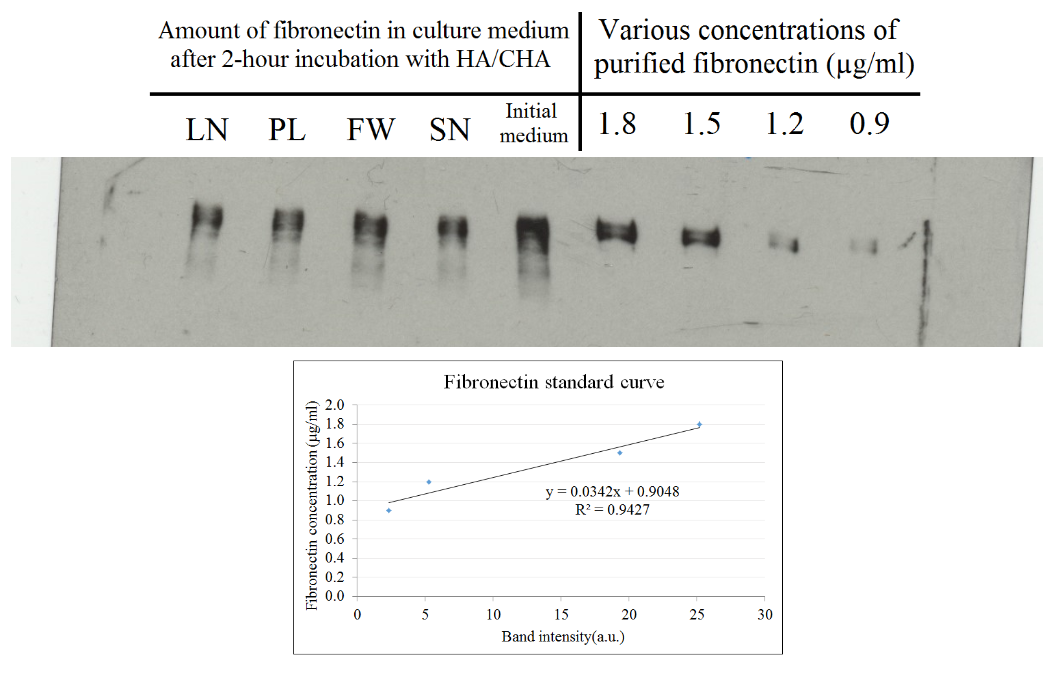


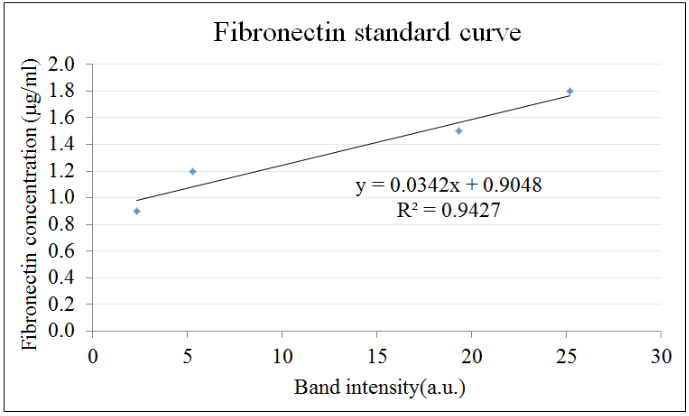


Original Fibronectin Blot Image


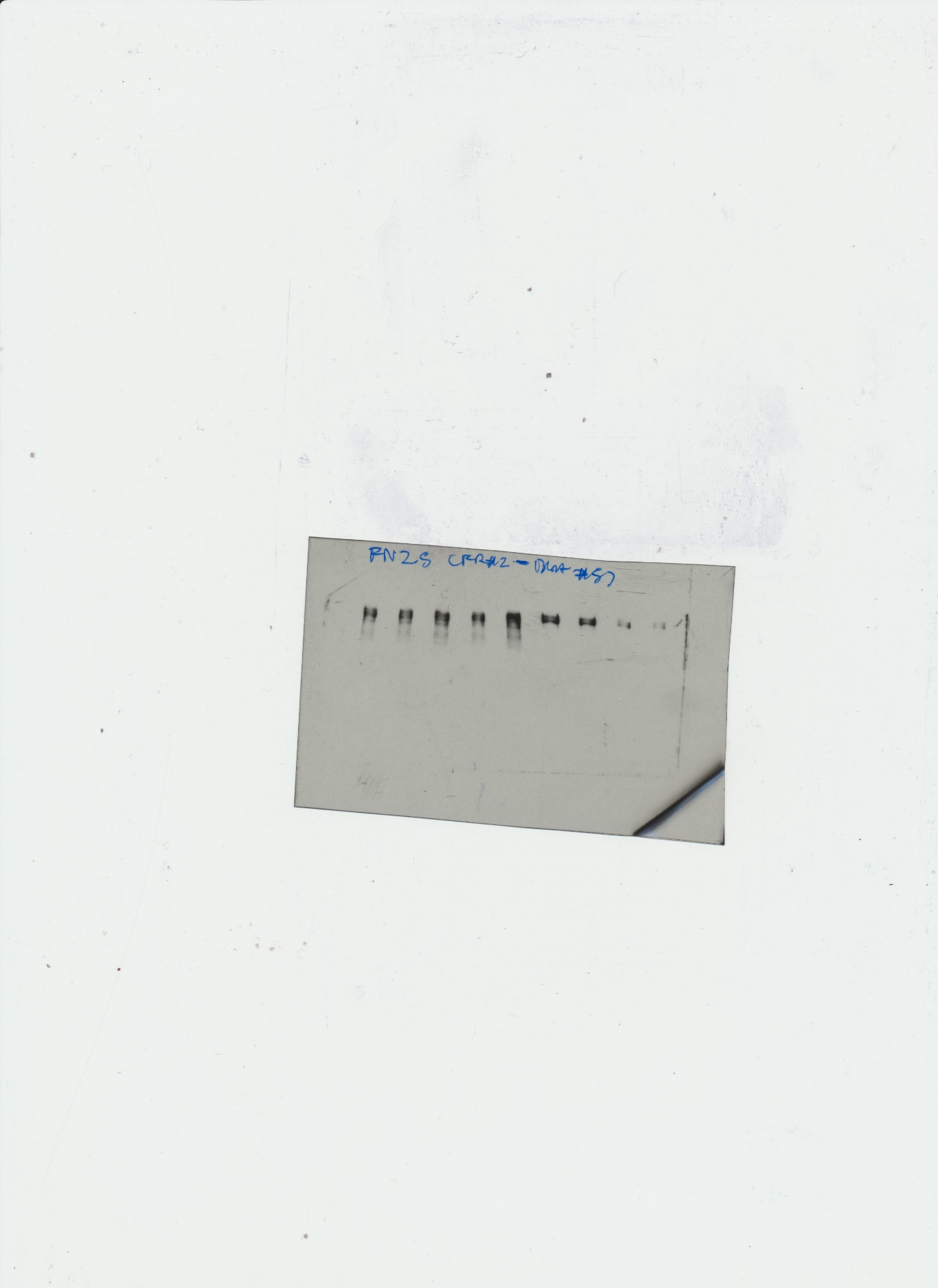


Western blot of fibronectin after 2-hour incubation with various HA/CHA microstructures with a standard curve constructed by known concentrations of purified fibronectin


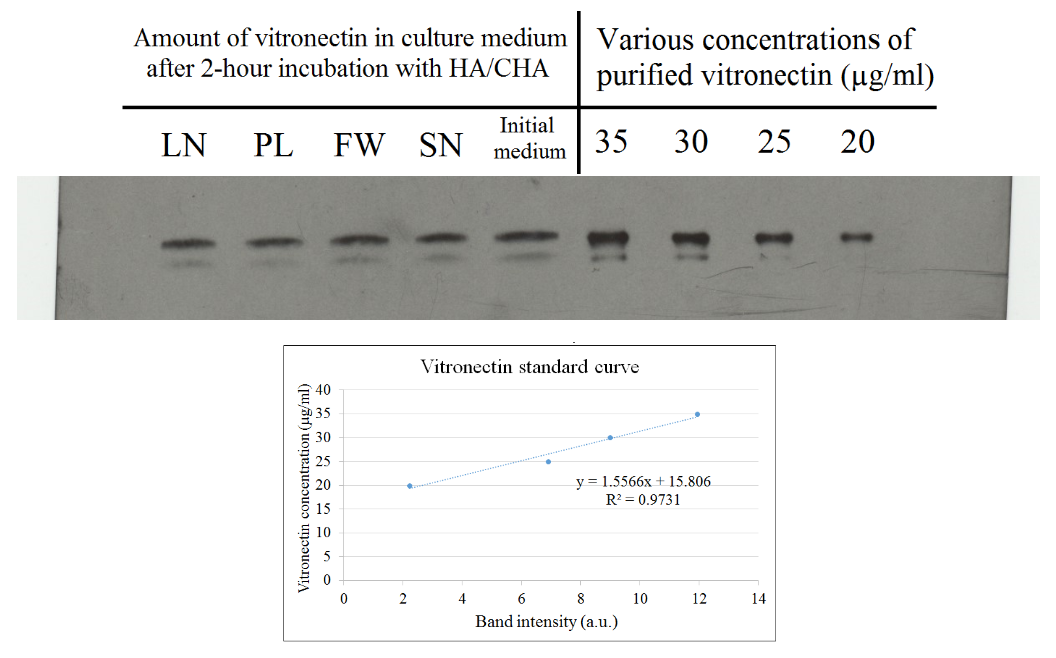


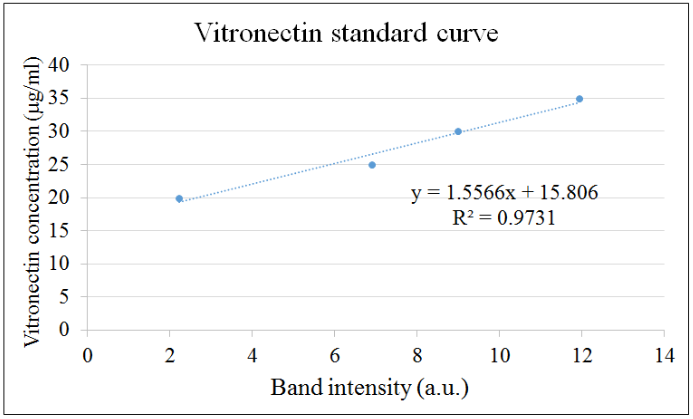


Original Vitronectin Blot Image


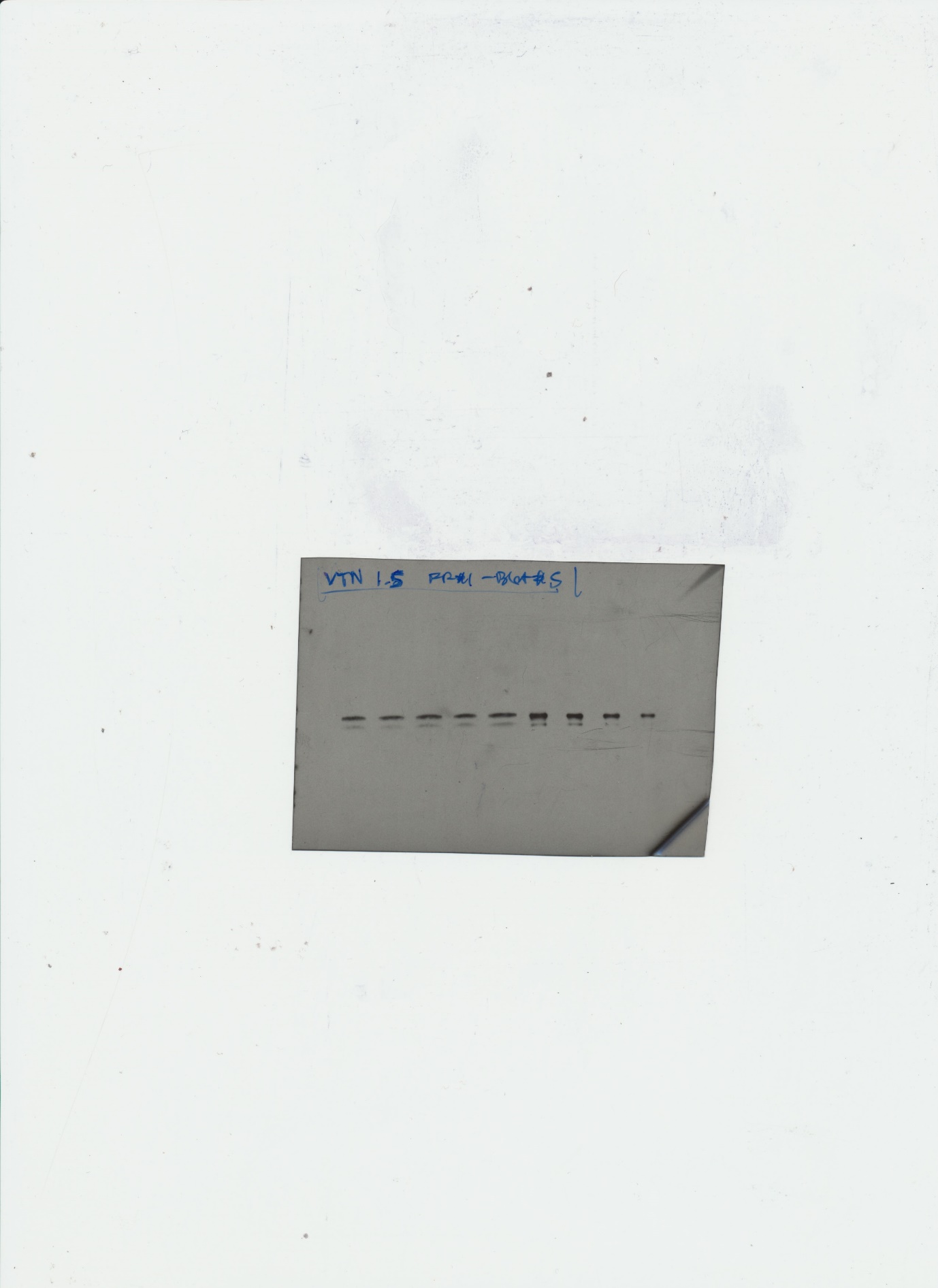


Western blot of vitronectin after 2-hour incubation with various HA/CHA microstructures with a standard curve constructed by known concentrations of purified vitronectin


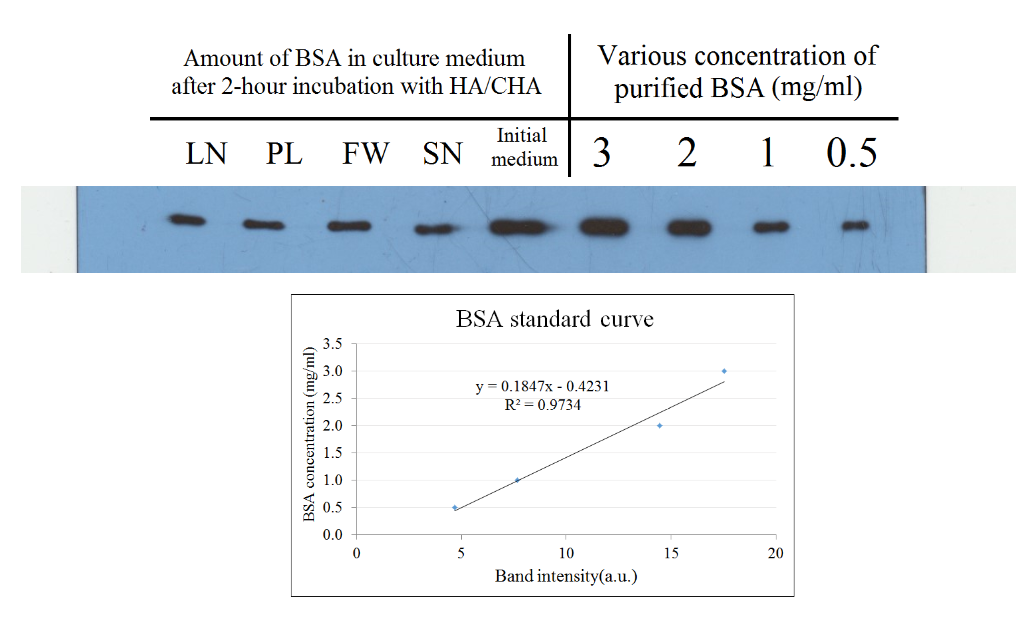


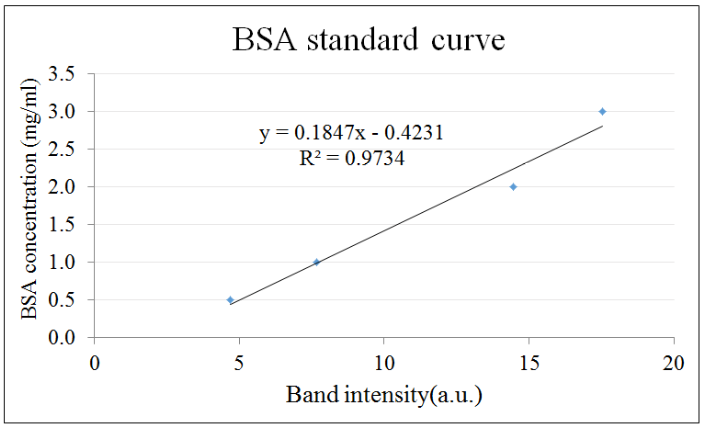


Original BSA Blot Image


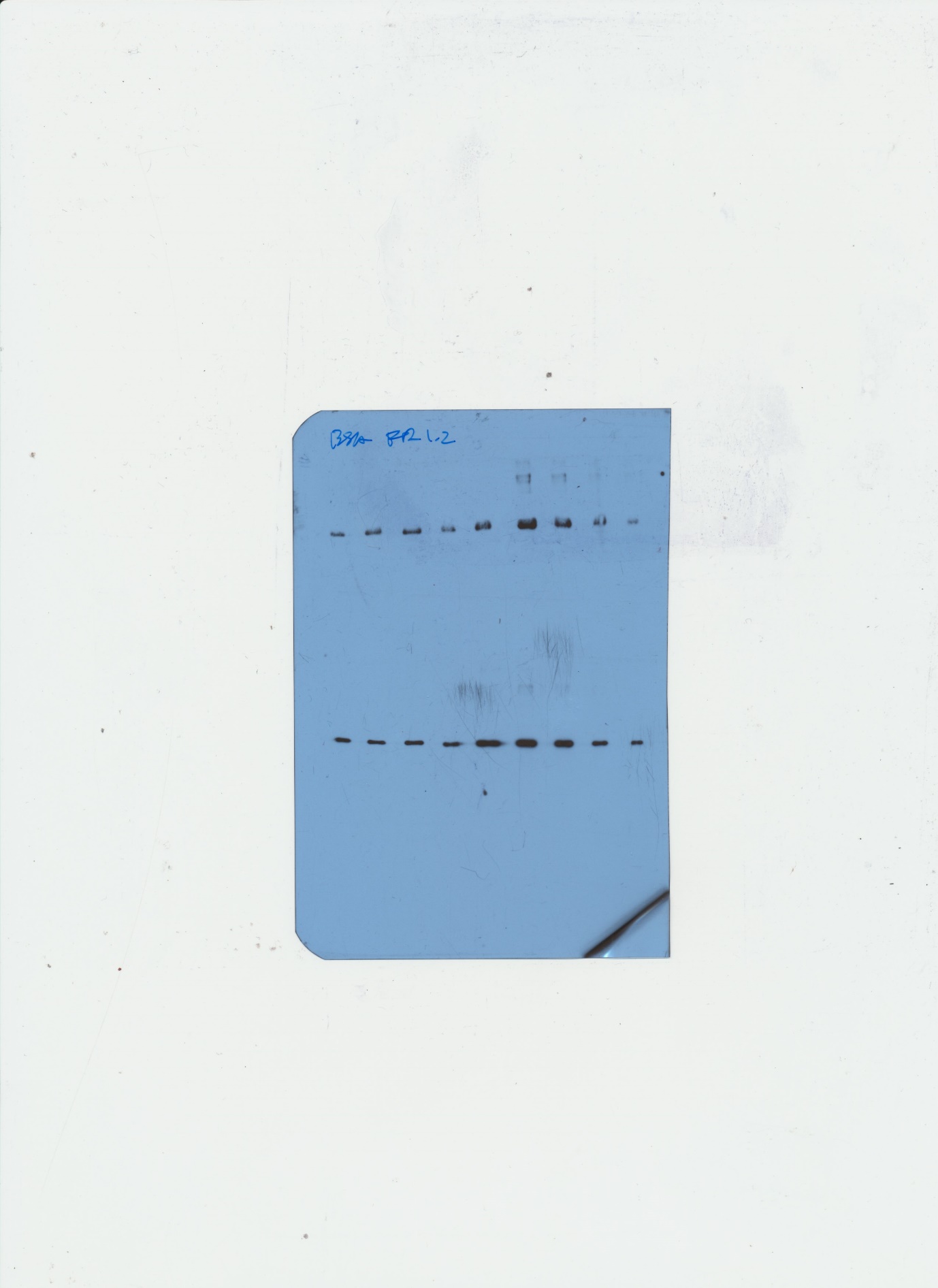


Western blot of BSA after 2-hour incubation with various HA/CHA microstructures with a standard curve constructed by known concentrations of purified BSA
